# A Reciprocal Link Between Gut Microbiota, Inflammation and Depression: A Place for Probiotics?

**DOI:** 10.3389/fnins.2022.852506

**Published:** 2022-04-25

**Authors:** Ahmed Eltokhi, Iris E. Sommer

**Affiliations:** ^1^Department of Pharmacology, University of Washington, Seattle, WA, United States; ^2^Department of Biomedical Sciences of Cells & Systems, University Medical Centre Groningen, University of Groningen, Groningen, Netherlands

**Keywords:** gut microbiota, inflammation, depression, sex hormones, probiotics

## Abstract

Depression is a severe mental disorder that places a significant economic burden on public health. The reciprocal link between the trillions of bacteria in the gut, the microbiota, and depression is a controversial topic in neuroscience research and has drawn the attention of public interest and press coverage in recent years. Mounting pieces of evidence shed light on the role of the gut microbiota in depression, which is suggested to involve immune, endocrine, and neural pathways that are the main components of the microbiota-gut-brain axis. The gut microbiota play major roles in brain development and physiology and ultimately behavior. The bidirectional communication between the gut microbiota and brain function has been extensively explored in animal models of depression and clinical research in humans. Certain gut microbiota strains have been associated with the pathophysiology of depression. Therefore, oral intake of probiotics, the beneficial living bacteria and yeast, may represent a therapeutic approach for depression treatment. In this review, we summarize the findings describing the possible links between the gut microbiota and depression, focusing mainly on the inflammatory markers and sex hormones. By discussing preclinical and clinical studies on probiotics as a supplementary therapy for depression, we suggest that probiotics may be beneficial in alleviating depressive symptoms, possibly through immune modulation. Still, further comprehensive studies are required to draw a more solid conclusion regarding the efficacy of probiotics and their mechanisms of action.

## Gut Microbiota in Health

The trillions of bacteria inhabiting our gastrointestinal (GI) tract are referred to as “gut microbiota” and are well known to exert a marked influence on the host during homeostasis (Thursby and Juge, 2017). Approximately 160 species of bacteria are living in the human colon, with *Bacteroidetes* and *Firmicutes* being the dominant bacterial phyla in healthy individuals (Qin et al., 2010; Huttenhower et al., 2012; Sommer and Backhed, 2013; Rajilic-Stojanovic and de Vos, 2014). The gut microbiota are separated from immune cells by two mucus layers and a single layer of epithelial cells (Johansson et al., 2013; Mowat and Agace, 2014), allowing the regulation of the immune system and vice versa (Round and Mazmanian, 2009; Gensollen et al., 2016). They benefit the host by strengthening the gut integrity (Natividad and Verdu, 2013), providing nutrients such as vitamins (LeBlanc et al., 2013; Rowland et al., 2018), promoting resistance to colonization by pathogenic species (Cameron and Sperandio, 2015; Pacheco and Sperandio, 2015; Sassone-Corsi and Raffatellu, 2015; Baumler and Sperandio, 2016) and harvesting energy (den Besten et al., 2013). Therefore, a pathological alteration of the gut microbiota composition, known as dysbiosis, can have serious consequences on the health of the host, ranging from chronic GI diseases to neuropsychiatric disorders (Guinane and Cotter, 2013; Petersen and Round, 2014; Schroeder and Backhed, 2016).

The high-throughput and low-cost sequencing methods that have become available over the last decade enabled the investigation of the gut microbiota (Thursby and Juge, 2017). The bacterial species are nowadays easily distinguished *via* targeting the bacterial 16S ribosomal RNA gene that is present in all bacteria with enough sequence conservation and nine highly variable regions for phylogenetic analyses (Mizrahi-Man et al., 2013; Poretsky et al., 2014). To characterize the nature of the human microbiota and pave the way for a better understanding of human health and disease, the Human Microbiome Project (HMP) was carried out over 10 years and two phases (Turnbaugh et al., 2007; Proctor et al., 2019). Additionally, it can identify new diagnostic biomarkers of health, which will enhance our knowledge of the nutritional requirements of humans (Turnbaugh et al., 2007).

**FIGURE 1:**
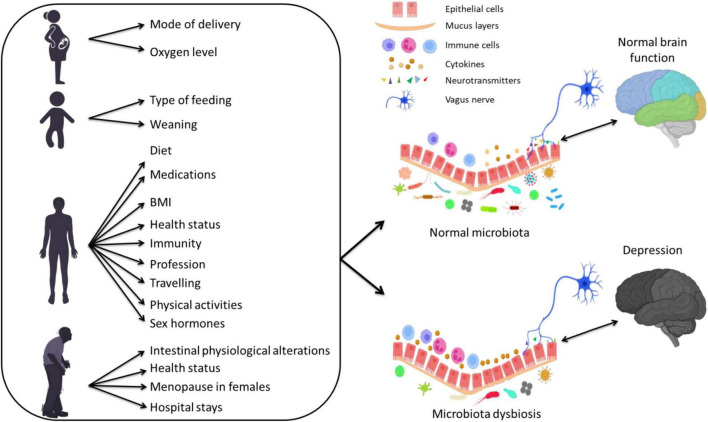
The effect of several factors during different stages of life on the gut microbiota composition and their association with depression *via* altering inflammatory responses.

Different factors are known to shape the gut microbiota compositions including age (Odamaki et al., 2016; Hill et al., 2017; Aleman and Valenzano, 2019), host genetics (Kovacs et al., 2011; Org et al., 2016; Elderman et al., 2018; Kim et al., 2020), drugs (Wilson and Nicholson, 2009; Maier et al., 2018; Vich Vila et al., 2020), body mass index (BMI) (Ley et al., 2005; Yun et al., 2017; Gao et al., 2018; Stanislawski et al., 2018; Bai et al., 2019), diet, mode of delivery (Salminen et al., 2004; Rutayisire et al., 2016; Akagawa et al., 2019; Reyman et al., 2019), and environmental factors (Li et al., 2014; Osadchiy et al., 2019) (Figure 1). In particular, dietary modification exert a large effect on the gut microbiota (Walker et al., 2011; Zoetendal et al., 2012; David et al., 2014; Carmody et al., 2015) and can produce a shift in several bacterial species within 24 h (Wu et al., 2011). Also, antibiotics affect microbiota composition by depleting resident microbiota and subsequent enrichment of certain antibiotic-resistant strains, leading to pathogenic effects (Morgun et al., 2015). Other non-antibiotic drugs, such as proton-pump inhibitors, but also several psychiatric drugs, are known to have an extensive impact on the human gut microbiota (Maier et al., 2018).

## Microbiota Dysbiosis in Depression

Depression is a debilitating neuropsychiatric disorder that involves persistent low mood and loss of interest that can be long-lasting or recurrent, substantially decreasing the quality of life (Wittchen et al., 2011; Lim et al., 2012; Murray et al., 2013; Whiteford et al., 2013). Symptoms usually are a reduced interest or pleasure in previously pleasurable activities, loss of sexual desire, changes in appetite, weight loss or gain, sleep disorders and motor retardation, along with recurrent thoughts of guilt and death (McCarter, 2008). It affects approximately 4.4% of the world’s population with an incidence rate above the rate of global population growth (Flux and Lowry, 2020). Despite the high prevalence rate of depression and the ongoing efforts to enhance the skills of healthcare providers, this mental disorder remains underdiagnosed and undertreated (Williams et al., 2017). The late diagnosis and treatment of depression are associated with a reduced treatment outcome, underscoring the importance of early intervention (Riedel et al., 2011).

The development of depression involves a complex interplay of biological, genetic, and environmental factors (Lopizzo et al., 2015). In addition to the strong association between gut microbiota and autism, schizophrenia and attention deficit hyperactivity disorder (for a review, see Eltokhi et al., 2020), accumulating shreds of evidence over the past 10 years support the hypothesis that gut microbiota may determine the initial risk and persistence of depression and contribute to treatment and resilience (for reviews, see Mangiola et al., 2016; Rogers et al., 2016; Flux and Lowry, 2020). The gut microbiota can signal to the CNS by way of neurohormones, vagal tonus, immune activation and metabolites that can alter eating behavior and mood (Martin et al., 2018). Moreover, healthy diets such as the Mediterranean one are suggested to reduce the risk of depression (Sanchez-Villegas et al., 2007; Skarupski et al., 2013; Pagliai et al., 2018; Konstantinos and Evangelia, 2019; Lassale et al., 2019) *via* modulation of the gut microbiota composition (De Filippis et al., 2016; Garcia-Mantrana et al., 2018; Krznaric et al., 2019; Nagpal et al., 2019). Confirming the role of gut microbiota in depression, a large epidemiological study revealed that the destabilization of the gut microbiota composition by antibiotics resulted in a 20–50% increased risk of depression (Lurie et al., 2015). Moreover, the underrepresentation of *Firmicutes* in the gut has been associated with depression (Huang et al., 2018), and 25.8% of individuals with inflammatory bowel disease (IBD) suffered from depression in the previous year (Lai and Cai, 2018).

Preclinical studies on rodents have investigated the link between gut microbiota and depression at a deeper level than clinical studies (Flux and Lowry, 2020). Studies mostly from the microbiota-devoid germ-free mice or mice treated with broad-spectrum antibiotics have shown that specific microbiota can impact brain physiology and behavior including deficiencies in learning, memory, recognition, and emotional behaviors (Gareau et al., 2011; Smith, 2015; Foster et al., 2017), accompanied by structural changes in the brain (Zhang et al., 2015; Principi and Esposito, 2016; Dinan and Cryan, 2017; Fung et al., 2017; Martin and Mayer, 2017). Different rodent models that showed high face validity to human depression have been used in microbiota research (Krishnan and Nestler, 2011). These models include olfactory bulbectomy (Harkin et al., 2003), social stress models (Toyoda, 2017), maternal separation (MS) models of early life adversity (Matthews and Robbins, 2003; Neumann et al., 2005; O’Mahony et al., 2009), repeated restraint stress models (Glavin et al., 1994; Bailey et al., 2010), chronic unpredictable mild stress (Duan et al., 2021; Zhang et al., 2021), and diet-induced obesity (Bruce-Keller et al., 2015; Bridgewater et al., 2017; Agusti et al., 2018; Soto et al., 2018) (for a review, see Flux and Lowry, 2020). The depression-like behaviors in rodents are measured by several behavioral experiments including forced swim, tail-suspension, sucrose preference, splash and learned helplessness tests (Eltokhi et al., 2018). Confirming the link between gut microbiota and depression, stressed germ-free mice showed high circulating levels of depression-sustaining hormones such as ACTH and corticosterone (Sudo et al., 2004). Moreover, the transplantation of fecal matter from depressed patients into microbiota-depleted rats exhibited depressive-like behavior in the rats (Kelly et al., 2016).

Clinical evidence for the role of the microbiota in depression is provided by an alteration in the number of microbiota and their diversity in individuals with depression when compared to healthy controls (for a summary, see Table 1). A study of fecal samples from patients with major depressive disorder (MDD) documented increased counts of *Bacteroidales* and decreased counts of *Lachnospiraceae* (Naseribafrouei et al., 2014). The analysis of fecal matters from MDD patients revealed a negative, albeit weak, correlation between the abundance of *Faecalibacterium* and the severity of the depressive symptoms (Jiang et al., 2015), and a positive correlation between *Enterobacteriaceae* and *Alistipes* counts and depression (Jiang et al., 2015). In another clinical study, lower *Bifidobacterium* and/or *Lactobacillus* counts were more common in patients with MDD compared to healthy controls (Aizawa et al., 2016). Two other studies revealed a different abundance of bacterial families and taxa between patients with MDD and healthy controls (Chen Z. et al., 2018; Chung et al., 2019). Moreover, in a Chinese MDD cohort, alterations of microbiota composition were found in MDD patients with an overrepresentation of *Actinobacteria* and underrepresentation of *Bacteroidetes* (Zheng et al., 2016). Investigating a group suffering from treatment-resistant MDD revealed a link between microbiota neuroactive capacity and the quality of life and depression (Valles-Colomer et al., 2019). Similar gut microbiota dysbiosis between Irritable bowel syndrome (IBS), depression, and IBS/depression co-morbid patients confirms the close link between the gut microbiota composition and depression (Liu et al., 2016). Although several previous studies demonstrated that depression was linked to marked alterations in gut microbiota composition, contradicting results regarding the enrichment of certain microbiota phyla in individuals with depression were obtained (Table 1). This inconsistency may have resulted from differences in study design, sample sizes, demographic and clinical severity of patients, unmeasured confounding factors and/or the used statistical methods. Additionally, most of these studies had several limitations including small sample sizes and a lack of data on the dietary habits of the tested subjects, which hinders obtaining a solid conclusion about the suitability of the microbiota as a biomarker of depression. It is still unsure whether changes in the microbiota are a cause or a result of depression. As depression is associated with changes in dietary habits, sleep and stress, the gut microbiota dysbiosis may well be a consequence rather than a cause. On the other hand, the depressive phenotype was shown to be transmissible by fecal microbiota transplantation (FMT) from MDD patients to germ-free rodents, revealing increased anxiety and depressive-like behavior in rodents (Kelly et al., 2016; Zheng et al., 2016). This strongly suggests that the alterations of the gut microbiota associated with depression may be a cause, rather than a consequence of the disorder.

**TABLE 1:** Clinical studies investigating a correlation between the human fecal microbiota and depression.

References	Sample	Sample size (patients/controls)	Mean age in years ± SD	Gender (males/females)	Changes in taxonomic composition in patients	Limitations/notes
Naseribafrouei et al., 2014	Fecal sample	55 Patients: 37 Controls: 18	Patients: 49.2 ± 13.9 Controls: 46.1 ± 13.9	Patients: (17/20) Controls: (7/11)	-High taxonomic level: ↑Bacteroidales ↓Lachnospiraceae -Low taxonomic level: ↑correlating OTUs of clades within the genus Alistipes and Oscillibacter	- A lack of data on the dietary habits of subjects - A small cohort with a risk of overseeing effects
Jiang et al., 2015	Fecal sample	76 Active-MDD: 29 Responded-MDD: 17 Controls: 30	Patients: Active-MDD: 25.3 ± 5.4 Responded-MDD: 27.1 ± 5.4 Controls: 26.8 ± 5.4	Patients: Active-MDD: (18/11) Responded-MDD: (9/8) Controls: (15/15)	-High taxonomic level: ↑Bacteroidete ↑Proteobacteria ↑Actinobacteria ↓Firmicutes -Low taxonomic level: ↑Enterobacteriaceae ↑Alistipes ↓Faecalibacterium	- A lack of data on the dietary habits of subjects - Possible effects of atypical antipsychotic medications - Further studies are required to evaluate the suitability of the microbiome as a biomarker.
Zheng et al., 2016	Fecal sample	121 Drug-naïve MDD: 39 Treated-MDD: 19 Controls: 63	Patients: 40.6 ± 11.7 Controls: 41.8 ± 12.3	Patients: (22/36) Controls: (23/40)	-High taxonomic level: ↑Actinobacteria ↓Bacteroidetes -Low taxonomic level: ↑OUTs assigned to the families Actinomycineae, Coriobacterineae, Lactobacillaceae, Streptococcaceae, Clostridiales incertae sedis XI (Parvimonas), Eubacteriaceae, Lachnospiraceae (Anaerostipes, Blautia, Dorea, Lachnospiracea incertae sedis), Ruminococcaceae (Clostridium IV) and Erysipelotrichaceae incertae sedis ↓OUTs assigned to the families Bacteroidaceae, Rikenellaceae (Alistipes), Lachnospiraceae (Coprococcus, Clostridium XlVa, Lachnospiraceae incertae sedis, Roseburia and Faecalibacterium), Acidaminococcaceae (Phascolarctobacterium), Veillonellaceae(Megamonas) and Sutterellaceae	- No examination of other neuropsychiatric disorders with similar clinical presentations to MDD - A possibility of site-specific and ethnic biases in microbial phenotypes
Aizawa et al., 2016	Fecal sample	100 Patients: 43 Controls: 57	Patients: 39.4 ± 10.0 Controls: 42.8 ± 12.7	Patients: (25/18) Controls: (22/35)	↓Bifidobacterium A tendency of ↓Lactobacillus	- Investigating only Bifidobacterium and Lactobacillus -Possible effects of antidepressant medications - Effects of diet were not fully taken into account in the analysis.
Chen Z. et al., 2018	Fecal sample	20 Patients: 10 Controls: 10	Patients: 43.9 ± 13.8 Controls: 39.6 ± 9.0	Patients: (5/5) Controls: (5/5)	-High taxonomic level: ↑Firmicutes ↑Actinobacteria ↓Bacteroidetes ↓Proteobacteria -Low taxonomic level: ↑Lachnospiraceae ↑Actinomycetaceae ↑Nocardiaceae ↑Bifidobacteriaceae ↑Erysipelotrichaceae ↑Clostridiaceae ↑Ruminococcaceae ↑Porphyromonadaceae ↑Streptomycetaceae ↓Enterobacteriaceae ↓Sutterellaceae ↓Oscillospiraceae ↓Chitinophagaceae ↓Marinilabiliaceae ↓Rikenellaceae ↓Prevotellaceae	- A limited sample size - No detailed data on diet habits, alcohol intake, or residence - Possible effects of antidepressant medications
Chung et al., 2019	Fecal sample	73 Patients: 36 Controls: 37	Patients: 45.83 ± 14.08 Controls: 41.19 ± 12.73	Patients: (8/28) Controls: (14/23)	-High taxonomic level: ↑Actinobacteria ↑Firmicutes ↓Bacteroidetes ↓Proteobacteria -Low taxonomic level: ↑Peptostreptococcaceae ↑Porphyromonadaceae ↑Streptococcaceae ↑Bifidobacteriaceae ↑Lachnospiraceae ↓Prevotellaceae ↓Alcaligenaceae	- A cross-sectional study design with no causal inference between the microbiota alterations and depression - A moderate sample size that provided no stable results with cluster analysis to further discuss the impacts of dietary patterns on microbiota compositions - Possible effects of antidepressant medications -The dietary information was subjective to recall bias.

*↑indicates an increase; ↓ indicates a decrease; MDD, Major depressive disorder; OTUs, Operational Taxonomic Units.*

Notably, several studies have shown that depression itself can affect the gut microbiota diversity *via* signals from the CNS to the gut environment, for example by changing secretion and motility in the stomach and gut. In an olfactory bulbectomy mouse model of depression, alteration of microbiota composition in the colon was suggested to be caused by increased activation of the stress response and alterations in colonic motility (Park et al., 2013). Moreover, diet disturbance in depression by the consumption of highly palatable foods is a major pathway from depression to gut microbiota dysbiosis (Rogers et al., 2016). Additionally, depression can reshape the gut microbiota composition by altering metabolic responses to food through hormones, inflammation, and autonomic alterations (Madison and Kiecolt-Glaser, 2019). For example, women suffering from depression had higher cortisol and fat oxidation levels (Kiecolt-Glaser et al., 2015), which can have a downstream effect on the gut microbiota (den Besten et al., 2013; Farzi et al., 2018). Other studies revealed that multiple classes of antidepressants also have anti-microbial properties against pathogenic bacteria (Macedo et al., 2017). For example, the antidepressant fluoxetine enriched the bacterial species associated with the regulation of BMI in mice (Lyte et al., 2019). The next-generation antidepressant ketamine plays a role in regulating the gut microbiota diversity (Yang et al., 2017) and showed increased *Lactobacillus*, *Turicibacter*, and *Sarcina* counts in the rat fecal microbiota and reduced opportunistic pathogens *Mucispirillum* and *Ruminococcus* (Getachew et al., 2018). To this end, the aforementioned studies suggest that the link between gut microbiota composition, antidepressants and depression is reciprocal.

## The Effect of Gut Microbiota on Local and Circulating Inflammatory Markers and Its Relation to Depression

Depression has been associated with inflammation and its components such as inflammatory cytokines for more than 20 years (Ader and Cohen, 1985; Maes, 1995; Maes et al., 2012; Patel, 2013; Skaper et al., 2014; Miller and Raison, 2016; Leonard, 2018). Several gene variants are involved in both immune activation and depression (Barnes et al., 2017). Moreover, depression is known to be associated with polymorphisms in inflammation-related genes (Mikova et al., 2001; Kokai et al., 2002; Wong et al., 2008). Elevated levels of pro-inflammatory cytokines including Interleukin (IL)-1, IL-6, IL-8, and IL-12 were reported in individuals suffering from depression (Dowlati et al., 2010; Duivis et al., 2013; Lamers et al., 2013; Kim et al., 2016). Furthermore, IL-6, tumor necrosis factor-alpha (TNF-α), and IL-1β are implicated in the pathophysiology of depression (Brebner et al., 2000), and individuals with depression often have elevated circulating IL-1β, IL-6, and TNF with reduced levels of interferon-gamma (IFN-γ), IL-10, and IL-4 (Goldsmith et al., 2016). Longitudinal studies linked the high levels of pro-inflammatory cytokines with future risk for depression (van den Biggelaar et al., 2007; Gimeno et al., 2009). The pro-inflammatory cytokines have been connected to a pattern of sickness behaviors that include depressed mood, lethargy, heightened pain sensitivity, and sleep and appetite disturbances: all hallmarks of depression (Flux and Lowry, 2020). Moreover, non-specific inflammatory markers such as haptoglobin, fibrinogen, and C-reactive protein (CRP) are increased in depressed patients (Sluzewska et al., 1996).

Supporting the relationship between inflammation and depression, significant inflammatory activity in rodent models of depression has been identified (Song and Wang, 2011). Moreover, several antidepressants have been shown to reduce the endogenous production of pro-inflammatory cytokines along with a modification of immune reactivity in the CNS (Capuron et al., 2002; Nazimek et al., 2017; Gałecki et al., 2018). Tricyclic antidepressants inhibit the release of pro-inflammatory cytokines IL-6, IL-1β, and TNF-α (Xia et al., 1996). Additionally, the next-generation antidepressant ketamine has been shown to decrease depressive symptoms *via* a decrease in circulating IL-1β levels (Tan et al., 2017), a protein known to cause neuroinflammation and depressive behaviors (Dunn et al., 2005; Dunn, 2006; Swiergiel and Dunn, 2007; Ransohoff and Brown, 2012). Conversely, anti-inflammatory drugs have been shown to enhance recovery in patients with depression (Mendlewicz et al., 2006; Muller et al., 2006), and depressive symptoms are observed clinically with the administration of IFNs (Baranyi et al., 2013; Mahajan et al., 2014). IL-10 knockout mice displayed a decreased latency to immobility in a forced swim test as a measure of depression, which was rescued by the administration of IL-10 (Mesquita et al., 2008) that is known to inhibit pro-inflammatory cytokine production (Ledeboer et al., 2000). Recent evidence indicated that microbiota dysbiosis is associated with the development of several chronic inflammatory disorders such as IBD (Ni et al., 2017). Notably, several gut microbial taxa with differential abundance patterns common to immune-mediated inflammatory diseases were identified (Forbes et al., 2018). Several genera including *Lactobacillus*, *Bifidobacterium*, and *Faecalibacterium* have been shown to stimulate the anti-inflammatory cytokine including IL-10 (Sokol et al., 2008) and downregulate inflammatory cytokines (Llopis et al., 2009).

As the gut microbiota play a fundamental effect on the gut inflammatory and immune responses, it is highly likely that the role of gut microbiota dysbiosis in the pathophysiology of depression is induced by immune and inflammation responses (Slyepchenko et al., 2017). Evidence of increased bacterial translocation due to the disruption of tight junctions and barrier integrity of the GI tract has now surfaced in the pathogenesis of depression (Slyepchenko et al., 2017). The non-invasive bacterial translocation to the mesenteric lymph nodes, the lamina propria, and the peripheral blood results in immune activation and increased production of pro-inflammatory cytokines. Heightened IgA and IgM-mediated immune responses to lipopolysaccharide, a component of the cell walls of gram-negative bacteria, in MDD support the notion of increased gut microbiota translocation due to a leaky gut (Maes et al., 2008).

**FIGURE 2:**
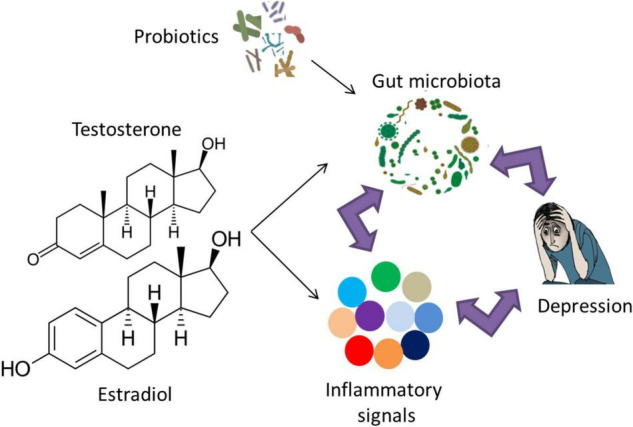
The reciprocal link between gut microbiota, inflammation and depression. The role of probiotic supplementation in turning the gut microbiota composition into a healthier form may lead to normal brain function and alleviation of depressive symptoms *via* modulation of inflammatory responses. Examples of probiotic genera include *Lactobacillus*, *Bifidobacterium*, *Lactococcus*, *Propionibacterium*, *Bulgaricus*, and *Streptococcus*. Notably, sex hormones affect gut microbiota and/or inflammatory signals, which may play a role in the sex bias of depression.

Evidence from rodent experiments confirms the link between microbiota and depression *via* inflammatory markers. Socially stressed mice showing increased fecal Oscillospira and a decreased fecal Firmicutes/Bacteroidetes ratio developed depression-like symptoms (Zhang et al., 2017). These symptoms were rescued *via* an intravenous treatment with an anti-mouse IL-6 receptor antibody (MR16-1) that significantly decreased *Oscillospira* counts and attenuated the decrease in the fecal *Firmicutes/Bacteroidetes* ratio (Zhang et al., 2017). Genetic deletion or pharmacological inhibition of Caspase-1 that cleaves IL-1β and IL-18 into their mature isoforms exhibited reduced depression- and anxiety-like behaviors in a chronic restraint stress model, combined with an increase in *Akkermansia* species that is associated with decreased inflammation and a rebalancing of the gut microbiota (Alcocer-Gomez et al., 2014; Anhe et al., 2015; Yang et al., 2015). Recent work has targeted the effects of the inflammasome in studying the relationship between the immune system, gut microbiota and depression (Inserra et al., 2018). It was hypothesized that the increased NLRP3 signaling due to stress/depression can lead to an overrepresentation of pro-inflammatory bacterial clades within the gut microbiota (Inserra et al., 2018). This pathological change in the gut microbiota composition may even strengthen the stress/depression phenotype and increase the risk of other NLRP3-related co-morbid disorders. Notably, several pieces of evidence have shown that the link between microbiota, inflammation and depression is indeed reciprocal (Figure 2). Microbiota dysbiosis in inflammatory diseases such as IBS can lead to depression (Tribbick et al., 2015; Liu et al., 2016). Conversely, repeated stress and/or depression increases pro-inflammatory signaling (Rohleder, 2014), leading to microbiota dysbiosis and increased representation of pro-inflammatory bacterial clades (Rogers et al., 2016; Wong et al., 2016; Zheng et al., 2016).

## Influence of Sex Hormones on the Gut Microbiota Composition and Gender Bias in Depression

Depression is more frequent in women than men with a ratio of 2:1, which was reported globally and independent of race or ethnicity (Weissman and Klerman, 1977; Cyranowski et al., 2000; Andrade et al., 2003; Ford and Erlinger, 2004; Patten et al., 2006; Bromet et al., 2011; Salk et al., 2017). The finding of similar female: male prevalence ratios worldwide suggests that the differential risk is highly dependent on biological sex differences rather than race, culture or other potentially confounding social and economic factors (Albert, 2015). As the onset of depressive disorders in women peaks in their reproductive years, the increased prevalence of depression may be explained, in part, by sex hormones. Indeed, the female hormonal fluctuation during puberty, menstruation, pregnancy, and menopause is a trigger for depression (Albert, 2015). Estrogen and progesterone affect neurotransmitter, neuroendocrine, and circadian systems that have been implicated in mood disorders (Wharton et al., 2012). Androgens seem to have anxiolytic properties, whereas estrogen receptor activation has opposite consequences, with ERα having largely anxiogenic-like properties and ERβ serving to generate anxiolytic-like effects (Borrow and Handa, 2017). Furthermore, rates of depression in females correlate with the low levels of estrogen that occur throughout the life cycle (Albert et al., 2015). The risk of depression appears to increase during the perimenopausal transition (Cohen et al., 2006), with hormone replacement therapy being effective in the prevention of postmenopausal depression in women (Gordon and Girdler, 2014). In this light, a study has indicated that women who reported using an oral contraceptive showed reduced rates of MDD compared with non-users (Cheslack-Postava et al., 2015), suggesting a protective role of estrogen against depression. In contrast, other studies have shown depression and worsened mood as potential adverse effects of hormonal contraceptive use (Wiebe et al., 2011; Gingnell et al., 2013; Skovlund et al., 2016; de Wit et al., 2020). The heterogeneity in these findings may be explained by differences in study populations (de Wit et al., 2020).

Current evidence confirming the role of sex hormones on depression incidence relies mainly on rodent work. A recent study has explored the effect of treatment with a 5-α-reductase inhibitor that converts steroid hormones testosterone and progesterone into dihydrotestosterone and dihydroprogesterone, respectively on depression-like behavior in rats, as well as 1 month of treatment withdrawal (Diviccaro et al., 2019). The withdrawal from the 5-α-reductase inhibitor was associated with elevated depression-like symptoms, as measured by the forced swim test (Diviccaro et al., 2019). Interestingly, a change in sex hormone concentration is associated with an altered immune profile and a shift in inflammation responses (Figure 2; Kupelian et al., 2009; Casimir et al., 2010; Karim et al., 2010; Maggio et al., 2011; Bobjer et al., 2013; Park and Lee, 2020; Grandys et al., 2021) (for reviews, see Monteiro et al., 2014; Au et al., 2016; Mohamad et al., 2018; Slavich and Sacher, 2019). The alteration of inflammatory responses due to sex hormones suggests their combined role in the pathophysiology of depression.

Although gut microbiota differed in males and females, castrated male mice showed similar microbiota composition to females, suggesting an influence of androgens on the gut microbiota composition (Markle et al., 2013; Yurkovetskiy et al., 2013). Moreover, testosterone treatment after gonadectomy rescued the alteration in the gut microbiota composition that was observed in the untreated males (Org et al., 2016). Several studies have shown a direct effect of estrogen on the gut microbiota composition (Flores et al., 2012; Cox-York et al., 2015; Chen and Madak-Erdogan, 2016; Org et al., 2016). Treatment with a 5-α-reductase inhibitor was associated with increased *Bacteroidetes* and *Prevotellaceae* counts, and the withdrawal was associated with a reduction in the family *Ruminococcaceae* and the genera *Oscillospira* and *Lachnospira* (Diviccaro et al., 2019). Interestingly, there seems to be a reciprocal interaction between gut microbiota and sex hormones leading to a change in the level of sex hormones because of differences in gut microbiota (Markle et al., 2013) and that gut microbiota regulate the production and/or utilization of testosterone and cause a difference in metabolism (Colldén et al., 2019).

Several studies have addressed the effects of sex on the gut microbiota in humans, which is suggested to be mediated, at least in part, by a difference in sex hormones levels (Figure 2; Mueller et al., 2006; Li et al., 2008; Ding and Schloss, 2014; Dominianni et al., 2015; Haro et al., 2016; Singh and Manning, 2016; Borgo et al., 2018; Gao et al., 2018; Sinha et al., 2019; Takagi et al., 2019) (for a recent review, see Kim et al., 2020). The sex differences in the gut microbiota composition mediated by sex hormones may exert adverse inflammatory and psychological effects related to depression and other neuropsychiatric disorders (Yurkovetskiy et al., 2013). For instance, adult women have reduced *Bacteroidetes* counts in their gut compared to age-matched men (Dominianni et al., 2015). Interestingly, the low levels of Bacteroidetes were previously observed in the fecal microbiota from patients diagnosed with clinical depression (Naseribafrouei et al., 2014). A study in a Chinese cohort revealed increased *Actinobacteria* levels in female MDD patients compared to female controls but reduced *Bacteroidetes* levels in male MDD patients compared to male controls (Chen J. J. et al., 2018).

In summary, the sex differences in the gut microbiota composition due to the effect of sex hormones may play a role in the sex bias of depression.

## Probiotics as an Adjunctive Therapeutic Option for Depression

Given the strong association between the gut microbiota and the pathophysiology of depression, modulation of the gut microbiota composition can be a promising method for ameliorating the behavioral symptoms related to depression along with other neuropsychiatric disorders (Larroya-Garcia et al., 2019). One way for maintaining a healthy gut microbiota composition is mediated by the supplementation of probiotics; the consumable microbes intended to promote a healthy microbiota and can provide a benefit to the host when administered in adequate amounts (Butel, 2014) (Figure 2). The main bacterial genera used as probiotics in preclinical and clinical studies are the *Lactobacillus* and *Bifidobacterium* genera (Genedi et al., 2019). Studies involving animal models demonstrated that probiotics improved cognition, mood, anxiety, and stress (Sudo et al., 2004; Desbonnet et al., 2010; Bravo et al., 2011; Ait-Belgnaoui et al., 2014; Smith et al., 2014; Mohle et al., 2016; Bruce-Keller et al., 2018; Chunchai et al., 2018; Hadizadeh et al., 2019). Probiotics have also been studied in non-psychiatric individuals, and initial work showed improvements in cognitive function (Marotta et al., 2019) along with reducing constipation in different populations (Chmielewska and Szajewska, 2010; Miller and Ouwehand, 2013; Dimidi et al., 2014). Probiotic supplementation showed an enhancement in sleep, autonomic balance, and bowel habits and reduced stress and cortisol levels in Japanese medical students (Nishida et al., 2017). Moreover, healthy women who drank a probiotic-containing fermented milk product for 1 month showed lower brain activation when exposed to emotional stimuli (Tillisch et al., 2013). In a randomized controlled trial in healthy individuals, multispecies probiotics were able to reduce cognitive reactivity toward sad moods *via* a reduction in rumination and aggressive thoughts (Steenbergen et al., 2015). The *Lactobacillus helveticus* and *Bifidobacterium longum* probiotic mix given to healthy individuals for 1 month alleviated psychological distress compared to a control group, similar to the results seen in rats (Messaoudi et al., 2011). Moreover, the healthy individuals in the bottom third on the depressed/elated dimension reported improved mood after drinking probiotic-containing milk for 3 weeks (Benton et al., 2007).

The desire for a more effective treatment and a prevention of depression seems perpetually at the top of the list in terms of global health concerns to reduce the burden of this condition. The idea of treating depression with probiotics roots back to 1910 when Dr. George Porter Phillips reported that a gelatin-whey formula comprised of lactic-acid-producing bacteria decreased depressive symptoms in melancholic adults (Phillips, 1910). Preclinical studies and clinical trials have increasingly investigated the roles of probiotics in the treatment of depressive-like behaviors. In rodents, there is considerable evidence suggesting the effect of probiotics in decreasing the depression-like behaviors. *Lactobacillus rhamnosus* administration reduced stress-induced anxiety- and depressive-like behaviors in mice (Bravo et al., 2011). *Bifidobacterium longum* improved the depressive-like phenotype during the tail suspension test in a mouse model of heightened anxiety (Savignac et al., 2014). Likewise, daily *Lactobacillus helveticus NS8* treatment reduced anxiety- and depressive-like behavioral dysfunctions in adult specific-pathogen-free rats facing chronic restraint stress in comparison to a selective serotonin reuptake inhibitor (SSRI) treatment (Liang et al., 2015). In a chronic mild stress model of depression, mice receiving a three-strain probiotic blend, *Lactobacillus helveticus*, *Lactobacillus plantarum*, and *Bifidobacterium longum*, displayed improved depression-like behavioral responses accompanied by a reduced TNF-α and interferon (IFN)-γ levels (Li et al., 2018). In another study, the administration of *Faecalibacterium prausnitzii via* oral gavage for 4 weeks in a chronic unpredictable mild stress model in rats resulted in decreased depression-like behaviors accompanied by increased IL-10 and decreased IL-6 and CRP levels (Hao et al., 2019). In another study, an administration of a probiotic formulation containing *Lactobacillus plantarum*, *Lactobacillus rhamnosus*, *Bifidobacterium lactis*, *Bifidobacterium breve*, and *Pediococcus pentosaceus* alleviated depressive-like behaviors in mice and decreased corticosterone level by restoring the gut microbiota composition (Liu et al., 2020). In Fischer and Long Evans rats subjected to maternal deprivation, a probiotic mixture composed of *Lactobacillus helveticus*, *Bifidobacterium longum*, *Lactococcus lactis*, and *Streptococcus thermophilus* reduced anxiety- and depression-like behaviors accompanied by a change in the levels of certain metabolites (Daugé et al., 2020).

Clinical studies revealed contradicting results regarding the efficiency of probiotics in decreasing depressive symptoms (for a summary, see Table 2). In an 8-week, randomized, double-blind, placebo-controlled clinical trial, a triple-strain probiotic mix, *Lactobacillus acidophilus*, *Lactobacillus casei*, and *Bifidobacterium bifidum*, resulted in improvements in depression scores in BDI in an MDD cohort (Akkasheh et al., 2016). Additionally, other beneficial metabolic effects were revealed including a significant reduction in inflammatory markers such as serum insulin, homeostasis model assessment of insulin resistance and serum hs-CRP (Akkasheh et al., 2016). Another 8-week, randomized, double-blind, placebo-controlled clinical trial examining the effect of a probiotic mix of *Lactobacillus helveticus* and *Bifidobacterium longum* on mild to moderate MDD reported a decrease in depression score in BDI (Kazemi et al., 2019). Interestingly, a randomized, placebo-controlled, double-blind study reported that daily *Lactobacillus rhamnosus* during pregnancy and into the post-partum period significantly decreased postnatal anxiety and depression scores (Slykerman et al., 2017). Another study testing the add-on effect of probiotic and prebiotic, the non-digestible plant-based carbohydrates that serve as nutrition for resident bacteria, on fluoxetine revealed a decrease in the score of hamilton rating scale for depression (HAM-D) compared to the placebo after 6 weeks of treatment (Ghorbani et al., 2018). One study provided petrochemical workers with either a probiotic capsule, probiotic yogurt, or conventional yogurt control, with both probiotic conditions resulting in a reduction of depressive symptoms on the general health questionnaire (GHQ) and depression anxiety and stress scale (DASS) scores as compared to control (Mohammadi et al., 2016). In another study, the efficacy of *Bacillus coagulans* administration on MDD in IBS patients was tested and revealed an improvement in HAM-D, Montgomery-Asberg Depression Rating Scale (MADRS), Center for Epidemiological Studies Depression Scale (CES-D) as well as reduced serum myeloperoxidase, an inflammatory biomarker (Majeed et al., 2018). In a recent open-label pilot study, a probiotic supplement containing *Lactobacillus helveticus* and *Bifidobacterium longum* administrated once per day for 8 weeks showed an improvement in affective clinical symptoms after 4 weeks, which was sustained till the end of the study (Wallace and Milev, 2021).

**TABLE 2:** Clinical trials on probiotic supplementation in individuals with depression.

References	Sample size (INT/PL)	Mean age in years ± SD	Gender (males/females)	Study compound and duration	A change in INT group	Limitations/notes
Akkasheh et al., 2016	MDD: 40 INT: 20 PL: 20	INT: 38.30 ± 12.10 PL: 36.20 ± 8.20	INT: (3/17) PL: (3/17)	- The study was performed for 8 weeks. - INT received probiotic capsules daily and consisted of *Lactobacillus acidophilus* (2 × 10^9^ CFU/g), *Lactobacillus casei* (2 × 10^9^ CFU/g), and *Bifidobacterium bifidum* (2 × 10^9^ CFU/g). - PL received capsules containing starch but no bacteria.	- ↓ BDI - ↓Serum insulin levels, homeostasis model assessment of insulin resistance, and serum hs-CRP concentrations - ↑Plasma total glutathione levels - No change in fasting plasma glucose, homeostatic model assessment of beta-cell function, quantitative insulin sensitivity check index, lipid profiles, or total antioxidant capacity levels	- No analysis of other biomarkers of inflammation or oxidative stress - A short intervention - Not knowing which strain in the probiotic supplements caused the treatment effect
Mohammadi et al., 2016	petrochemical workers: 70 INT 1: 25 INT 2: 25 PL: 20	INT 1: 33.20 ± 6.40 INT 2: 31.50 ± 5.80 PL: 33.10 ± 6.10	INT 1: (12/13) INT 2: (12/13) PL: (12/8)	- The study was performed for 6 weeks. - INT 1 received 100 g/day probiotic yogurt (*Lactobacillus acidophilus* LA5 and *Bifidobacterium lactis* BB12 with a total of min 1 × 10^7^ CFU) + one placebo capsule. - INT 2 received one probiotic capsule daily (*Lactobacillus casei* 3 × 10^3^, *Lactobacillus* acidophilus 3 × 10^7^, *Lactobacillus rhamnosus* 7 × 10^9^, *Lactobacillus bulgaricus* 5 × 10^8^, *Bifidobacterium breve* 2 × 10^10^, *Bifidobacterium longum* 1 × 10^9^, *Streptococcus thermophilus* 3 × 10^8^ CFU/g and 100 mg fructo-oligosaccharide with lactose as carrier substances) + 100 g/day conventional yogurt. - PL received 100 g/day conventional yogurt (*Streptococcus thermophilus* and *Lactobacillus bulgaricus*) + one placebo capsule (the same substance without bacteria).	- ↑GHQ in INT 1 and INT 2 - ↓DASS scores in INT 1 and INT 2 - No change of GHQ or DASS scores in PL	- A short intervention -No assessment of the rate that short-chain fatty acids were produced by probiotics in the gut
Slykerman et al., 2017	Pregnant women of 14–16 weeks gestation: 423 INT: 212 PL: 211	INT: 33.50 ± 4.24 PL: 33.70 ± 4.44	Only females	- The study was performed until 6 months postpartum if breastfeeding. -INT received a daily *Lacticaseibacillus rhamnosus* HN001 at a dose of 6 × 10^9^ CFU. -PL received a daily placebo (corn-derived maltodextrin).	- ↓Depression and anxiety scores in EPDS and STAI6 - ↓Clinically relevant anxiety on screening	- The EPDS and STAI6 are screening tools for postnatal depression and anxiety, but not diagnostic. -The information was collected retrospectively, and neither the EPDS nor the STAI6 has been validated using the questions phrased in the past tense.
Ghorbani et al., 2018	Individuals with moderate depression: 40 INT: 20 PL: 20	INT: 34.45 ± 3.95 PL: 35.50 ± 5.27	INT: (6/14) PL: (6/14)	- All Patients received fluoxetine (20 mg/d) for 4 weeks before and throughout the whole study. - The study was performed for 6 weeks. - INT received 2 capsules daily containing 500 mg probiotics (*Lactobacillus casei* 3 × 10^8^ CFU/g, *Lactobacillus acidophilus* 2 × 10^8^ CFU/g, *Lactobacillus bulgaricus* 2 × 10^9^ CFU/g, *Lactobacillus rhamnosus* 3 × 10^8^ CFU/g, *Bifidobacterium breve* 2 × 10^8^ CFU/g, *Bifidobacterium longum*, 1 × 10^9^ CFU/g, *Streptococcus thermophilus* 3 × 10^8^ CFU/g) and 100 mg fructooligosaccharide as prebiotic. - PL received 2 placebo capsules daily containing 1,000 mg magnesium stearate.	- ↓HAM-D score at the endpoint of the intervention	- A short period of follow-up - A small number of participants
Majeed et al., 2018	MDD: 40 INT: 20 PL: 20	INT: 40.36 ± 10.28 PL: 43.88 ± 9.85	INT: (3/17) PL: (3/17)	- The study was performed for 90 days. - INT received a daily dose of 2 × 10^9^ CFU *Bacillus coagulans* MTCC 5856 (600 mg) that contained also, microcrystalline cellulose, starch, sodium starch glycolate and magnesium stearate. -PL received placebo tablets that had the same ingredients except for *Bacillus coagulans* MTCC 5856.	- ↓HAM-D, MADRS, CES-D, IBS-QOL in INT - ↓CGI-I, CGI-S, Dementia – TFS, GI-DQ, mESS in INT - ↓ Serum myeloperoxidase in INT but not PL	- A short period of follow-up - A small number of participants
Kazemi et al., 2019	MDD: 110 INT 1: 38 INT 2: 36 PL: 36	INT 1: 36.15 ± 7.85 INT 2: 37.35 ± 7.97 PL: 36.00 ± 8.47	INT 1: (11/27) INT 2: (9/27) PL: (12/24)	- The study was performed for 8 weeks. - INT 1 received probiotic product consisting of freeze-dried *Lactobacillus helveticus* R0052 and *Bifidobacterium longum* R0175 (CNCM strain I-3470) bacteria (10 × 10^9^ CFU) per 5 g sachet + xylitol, maltodextrin, plum flavor and malic acid - INT 2 received a prebiotic product consisting of galactooligosaccharide and 0.2% Plum flavor - PL received a product consisting of xylitol, maltodextrin, plum flavor and malic acid.	- ↓BDI score in INT 1 compared to PL - ↓Kynurenine/tryptophan ratio compared to PL - ↑Tryptophan/isoleucine ratio compared to PL - No change of BDI score in INT 2 compared to PL - ↓Tryptophan/BCAAs ratio in INT 2 compared to PL	- 10 subjects from INT 1, 9 from INT 2 and 10 from PL dropped out before the trial completion - A lack of fecal microbiome analysis - The intervention was conducted at different times of the year - No control of changes in lifestyle, diet, vitamin D status, etc. - Different used antidepressant drugs
Wallace and Milev, 2021	MDD: 10 INT: 10	INT: 25.20 ± 7.00	INT: (3/7)	- The study was performed for 8 weeks. - INT received a probiotic supplement containing *Lactobacillus helveticus* R0052 and *Bifidobacterium longum* R0175 at a dose of 3 × 10^9^ CFU once per day.	- ↓MADRS, QIDS-SR16 and SHAPS scores - ↓GAD-7 and STAI scores - ↑Sleep quality measured by PSQI	-A small number of participants - An open-label design and lack of placebo - A reduced generalizability of the results - The sample was disproportionately skewed toward young adult females.
Östlund-Lagerström et al., 2016	General older adults: 249 INT: 125 PL: 124	INT: 72.60 ± 5.80 PL: 72.00 ± 5.60	INT: (54/71) PL: (43/81)	-The study was performed for 12 weeks. -INT received a product consisting of a stick-pack containing freeze-dried *Lactobacillus reuteri* DSM 17938 (1 × 10^8^ CFU/stick-pack), rhamnose, galactooligosaccharide and maltodextrin to a total weight of 1 g. -PL received a product consisting of maltodextrin.	- No change in GSRS, depression or anxiety scores - No change in the stress level (PSS scores) - No change in EQ-VAS or EQ-5D-index scores assessing the quality of life - No change in stool frequency	- A possibility that participants suffering from IBS were included in the study - An additional more fine-tuned instrument such as the ROME III symptom criteria, might have increased the sensitivity - The use of a low dose of *Lactobacillus reuteri* - The study was underpowered.
Romijn et al., 2017	Individuals with low mood: 79 INT: 40 PL: 39	INT: 35.80 ± 14.00 PL: 35.10 ± 14.50	INT: (8/32) PL: (9/30)	- The study was performed for 8 weeks. -INT received a product consisting of freeze-dried *Lactobacillus helveticus* R0052 and *Bifidobacterium longum* R0175 bacteria at a dosage of 3 × 10^9^ CFU per 1.5 g sachet + xylitol, maltodextrin, plum flavor and malic acid. -PL received a product consisting of xylitol, maltodextrin, plum flavor and malic acid.	- No change in MADRS, CGI-S, CGI-I, QIDS-SR16, GAF, DASS - No difference in biomarker levels	- No measurement of BMI, body fat, dietary intake and physical activity -A lack of intestinal microbiome analysis -A small sample size - A short length of the intervention period
Rudzki et al., 2019	MDD: 60 INT: 30 PL: 30	INT: 39.13 ± 9.96 PL: 38.90 ± 12.00	INT: (7/23) PL: (10/20)	- The study was performed for 8 weeks. -Both INT and PL received SSRI treatment during the whole study. - INT received 2 capsules daily with each containing 10 × 10^9^ CFU of probiotic bacteria *Lactobacillus Plantarum* 299v. - PL received 2 capsules daily containing crystalline cellulose powder.	- No change in HAM-D 17, SCL-90 or PSS - Improvement of work speed in APT - Improvement of CVLT total recall -↓Kynurenine concentration -↑3HKYN:KYN ratio	-A small sample size - No measurements of intestinal permeability - No measurement of QUIN or vitamin B levels
Reininghaus et al., 2020	Inpatient MDD: 61 INT: 28 PL: 33	INT: 43.00 ± 14.31 PL: 40.11 ± 11.45	INT: (8/20) PL: (6/27)	- The study was performed for 4 weeks. -INT and PL received 125 mg D-Biotin. -INT received probiotics containing *Bifidobacterium bifidum* W23, *Bifidobacterium lactis* W51, *Bifidobacterium lactis* W52, *Lactobacillus acidophilus* W22, *Lacticaseibacillus casei* W56, *Lacticaseibacillus paracasei* W20, *Lactiplantibacillus plantarum* W62, *Lactobacillus salivarius* W24 and *Lactococcus lactis* W19 + 30 mg of common horsetail, 30 mg of fish collagen and 30 mg of keratin plus matrix -PL received a product containing 30 mg of common horsetail, 30 mg of fish collagen and 30 mg of keratin plus matrix.	- No difference between INT and PL in any of the psychiatric scales - No difference in intestinal barrier function -↑*Ruminococcus gauvreauii* in INT -↑Taxonomically related *Coprococcus*	- A short length of the intervention Period -The strong decrease in depression in both groups may have masked the difference between them - Changes in nutritional habits might have influenced the results. -A difference at baseline for smoking status between both groups - The high number of females may have skewed the results.

*The clinical studies of Östlund-Lagerström et al. (2016), Romijn et al. (2017), Rudzki et al. (2019), Reininghaus et al. (2020) did not reveal a significant improvement in the depression phenotype. INT, Intervention; PL, Placebo; CFU, Colony-forming units, SSRI, Selective serotonin reuptake inhibitor; BDI, Beck Depression Inventory; GHQ, General health questionnaire; DASS, Depression anxiety and stress scale; EPDS, Edinburgh Postnatal Depression Scale; STAI6, State Trait Anxiety Inventory 6 item version; HAM-D, Hamilton rating scale for depression; MADRS, Montgomery–Asberg Depression Rating Scale; CES-D, Center for Epidemiological Studies Depression Scale; IBS-QOL, Irritable Bowel Syndrome Quality of Life Questionnaire; CGI-I, Clinical Global Impression-Improvement rating Scale; CGI-S, Clinical Global Impression Severity Rating Scale; Dementia – TFS, Dementia – Total frequency scoring; GI-DQ, Gastrointestinal Discomfort Questionnaire; mESS, Modified Epworth Sleepiness Scale; QIDS-SR16, Quick Inventory of Depressive Symptomatology; SHAPS, Snaith-Hamilton Pleasure Scale; GAD-7, Generalized Anxiety Disorder 7-item scale; STAI, State Trait Anxiety Inventory; PSQI, Pittsburgh Sleep Quality Index; GSRS, Gastrointestinal symptoms rating scale; PSS, Perceived stress scale; GAF, Global Assessment of Functioning; SCL-90, Symptom Checklist; APT, Attention and Perceptivity Test; CVLT, California Verbal Learning Test.*

Other studies in the literature failed to show any improvement of depression scores in MADRS following 8 weeks of primary treatment with probiotics mix of *Lactobacillus helveticus* and *Bifidobacterium longum* (Romijn et al., 2017). Moreover, there was no change in psychological symptoms and the concentrations of CRP, IL-1β, IL-6, or TNF between baseline and the end of the study (Romijn et al., 2017). Worth notice is that individuals in this study were not taking any antidepressant medication at the time of the study, and their depressive history was entirely self-reported without a formal diagnostic interview (Romijn et al., 2017). In another study of individuals diagnosed with MDD, SSRI treatment supplemented with *Lactobacillus plantarum* did not exhibit any improvement in depressive symptoms and did not change the levels of circulating TNF, IL-6, or IL-1β, although there was an increase in cognitive functioning (Rudzki et al., 2019). In another study, the health properties of *Lactobacillus reuteri* DSM17938 were investigated in individuals older than 65 and revealed no improvement in digestive health, general wellbeing, stress, anxiety, or depression (Östlund-Lagerström et al., 2016). In a randomized clinical study in 2020, a combination of 9 bacterial species *Bifidobacterium bifidum* W23, *Bifidobacterium lactis* W51, *Bifidobacterium lactis* W52, *Lactobacillus acidophilus* W22, *Lactobacillus casei* W56, *Lactobacillus paracasei* W20, *Lactobacillus plantarum* W62, *Lactobacillus salivarius* W24, and *Lactobacillus lactis* W19 in addition to biotin yielded no improvement in psychiatric symptoms in patients with MDD (Reininghaus et al., 2020).

## Conclusion

Gut microbiota were incorporated into neurobiological models of depression *via* an effect on immune, endocrine, and nervous system responses, allowing a more comprehensive model of depression (Figure 2). The strong association between microbiota dysbiosis and depression could pave the way for enhancing diagnostic accuracy and patient phenotyping for treatment selection. Moreover, it can advance the treatment and prevention of depression by modifying the gut microbiota composition and offer an important future strategy in psychiatry *via* nutritional interventions, prebiotic or probiotic supplementations. Although preclinical mechanistic experimental data indicated that the manipulation of the gut microbiota with probiotics may have antidepressant and anxiolytic effects, there is limited clinical evidence for the efficacy of probiotics in depression at present. Notably, several probiotic studies that failed to show an improvement in depression revealed also no change in pro-inflammatory markers. Given the reciprocal link between microbiota, inflammation and depression discussed in this review, we believe that probiotics may partially exert their roles through a modification of the immune system. Generally, additional randomized clinical trials in patients with depression are necessary to fully evaluate their therapeutic potential on the clinical diagnosis and inflammatory biomarkers. Different factors should be kept into consideration to maximize the benefit of probiotic supplementation. The microbial diversity obtained by incorporating multiple strains of organisms is suggested to be more effective than using a single organism. Investigations at a finer taxonomic level coupled with multi-omic techniques such as transcriptomics, proteomics, and metabolomics are also needed to exclude confounding factors as much as possible. Since sex hormones can affect the inflammation biomarkers, with probiotics causing different inflammatory responses in female and male mice (Karunasena et al., 2014; Lee et al., 2017), sex should be taken into account in studies on gut microbiota, probiotics and depression.

## Author Contributions

AE conceptualized and wrote the first draft. IES edited and reviewed the final draft. Both authors contributed to the article and approved the submitted version.

## Conflict of Interest

The authors declare that the research was conducted in the absence of any commercial or financial relationships that could be construed as a potential conflict of interest.

## Publisher’s Note

All claims expressed in this article are solely those of the authors and do not necessarily represent those of their affiliated organizations, or those of the publisher, the editors and the reviewers. Any product that may be evaluated in this article, or claim that may be made by its manufacturer, is not guaranteed or endorsed by the publisher.
